# Missing incidents and the risk of harm in persons living with dementia reported to the Swedish police– A nationwide retrospective registry study

**DOI:** 10.1186/s12877-025-05809-9

**Published:** 2025-03-05

**Authors:** Mikael Larsson, Kristofer Årestedt, Anders Svensson, Henrik Andersson, Maria Wolmesjö

**Affiliations:** 1 Faculty of Caring Science, Work Life and Social Welfare, University of Borås, Borås, Sweden; 2 Police Inspector at the Swedish Police Authority, Stockholm, Sweden; 3 Faculty of Health and Life Sciences, Linnaeus University, Kalmar, Sweden; 4 Department of Research, Region Kalmar County, Kalmar, Sweden; 5 Emergency Care, Centre of Interprofessional Collaboration within Emergency care (CICE), RN, PEN, Faculty of Health and Life Sciences, Linnaeus University, Växjö, Sweden; 6 Emergency Care, Centre for Prehospital Research (PreHospen), RN, Faculty of Caring Science, Work Life and Social Welfare, University of Borås, Borås, Sweden; 7 SOLWE, Faculty of Caring Science, Work Life and Social Welfare, University of Borås, Borås, Sweden

**Keywords:** Alzheimer disease, Dementia, Patient harm, Lost, Missing person, Police, Quantitative method, Regression analysis, Risk assessment, Wandering behavior

## Abstract

**Background:**

The number of persons living with dementia is increasing globally, including in Sweden, and these persons are at heightened risk of going missing and coming to harm. When they do go missing, the police get involved. There is a dearth of knowledge surrounding the prevalence and outcomes of harm in these instances in many countries, including Sweden, which affects our understanding of the associated risks and necessary interventions. Therefore, the aim of this study was to describe incidents of missing people and explore factors associated with harm in persons living with dementia as reported to the Swedish Police.

**Methods:**

Data on background characteristics, the missing incidents, and police response was collected from a nationwide police registry. The missing incidents were analysed using descriptive statistics and logistic regression was used to explore factors associated with harm.

**Results:**

A total of 1,041 missing person case reports concerning persons living with dementia were identified. In 61 (6%) of these reports, the missing person was harmed. The level of harm varied from lacerations to death. Male sex, no prior missing incidents, cold season, time since last contact, delayed reporting, and prolonged duration of police search effort were significantly associated with an increased probability of harm.

**Conclusions:**

Persons living with dementia constituted a substantial proportion of all missing persons case reports submitted to the Swedish Police during the study period. Persons living with dementia were also at considerable risk of harm when missing, as even minor injuries can lead to substantial consequences. Furthermore, time was a critical factor, emerging as the strongest predictor of harm in the study. This calls for the development of collaborative routines between the police and professional caregivers caring for persons living with dementia.

## Background

Persons living with dementia are at an increased risk of going missing and becoming lost [1–3]. When they do, they also face an elevated risk of harm [4], and in the worst cases, death [5]. However, knowledge about such missing incidents is still not well established [6] and neither is knowledge of harmful outcomes in such incidents [7]. When a person living with dementia goes missing, the police are often responsible for finding them.

Internationally, as well as in Sweden, one of the biggest welfare challenges to confront is the challenge of an aging population, including a growing population of persons living with cognitive impairments, or *dementia*, which here is used as a concept to summarize different diagnoses in this field [8]. Dementia is a primary contributor to impairment and dependence worldwide. The World Health Organization estimates that 55 million people were living with dementia worldwide in 2023. The number is estimated to grow with 10 million cases annually [9]. Sweden has a population of approximately 10.5 million people [10]. Among them, an estimated 150,000 are living with dementia. By 2050, this number is projected to rise to around 250,000 [11]. It is estimated that 97.5% of persons living with dementia in Sweden are 60 years or older, with 60% living in ordinary housing in the community and 40% in some kind of special housing for older adults, with access to social and healthcare 24 h a day, seven days a week [8, 12]. Internationally, the proportion of persons living with dementia in long-term care facilities varies. In the United States, fewer than half of nursing home residents have a formal dementia diagnosis, even though the proportion of individuals living with dementia in these settings is estimated to be significantly higher [13]. In the United Kingdom, 70% of care home residents are estimated to be living with dementia or severe memory problems [14]. Similarly, data from Canada and Australia show that 42% of individuals aged 80 and older in long-term care and 54% of those in permanent residential aged care, respectively, are living with dementia [15, 16].

Dementia is a condition that affects many aspects of life of those living with dementia, their relatives, the healthcare sector, the care of older people sector, rescue services, and the police [17]. One such aspect is the increased risk of going missing [1, 18].

Most persons living with dementia go missing during short periods of unsupervised activity while conducting their normal routines. These incidents are typically unpredictable, as they occur without any clear preceding signs [19, 20]. Most missing incidents are never reported to the police, leading to significant underreporting. Contacting the police is generally regarded as a last resort by caregivers [21, 22]. There has been insufficient focus on dementia-related symptoms and the risk of going missing as well as and the potential harm that may occur while missing [23, 24]. Studies on harm outcomes in missing incidents, regardless of age, gender, or cause, showed that 4% of missing persons were found harmed [25, 26]. When Doyle and Barnes [26] extracted data from two police districts in England involving persons aged 65 or older, the proportion of harmful outcomes increased to 7%. Different research methods, varying sample sizes, registry data, and diverse outcome measures have led to broad findings that are difficult to compare when it comes to harm associated with missing incidents in persons living with dementia [27]. For example, Koester and Stooksbury [28] found that 19% of missing persons living with dementia subject to a search and rescue operation in Virginia, United States, were found deceased; 69% experienced significant adverse outcomes, such as being in dangerous environments, exhibiting poor general condition, or suffering from dehydration or hypothermia; while 20% were found unharmed and were able to be escorted back to safety. In contrast, Murata et al. [24] found in a large quantitative study from Japan that 3% were found deceased, yet no other harm outcomes was studied. When missing persons living with dementia were harmed, lacerations, trauma related to falls or traffic accidents, dehydration and hypothermia were most common. Drowning was the most common cause of death [4, 24, 28–30].

Internationally, including in Sweden, the police are often responsible for locating missing persons at risk of harm [31, 32]. There is a knowledge gap on how to handle missing incidents in persons living with dementia within the police [3, 33]. A police search effort is a collective term for all actions undertaken by the police with the goal of locating a person who has been reported missing. It may or may not involve the dispatch of police patrols. Search and rescue operations are included within the term search effort but are typically more extensive and more organized forms of such efforts [32]. Research on police involvement in missing incidents concerning persons living with dementia is limited internationally and represents a new field of study in Sweden [33, 34]. There are about 25,000 missing incidents reported to the Swedish police each year. Based on a modest number of search and rescue reports, persons living with dementia have been estimated to constitute 20% of the missing incidents [33]. The Swedish Police do not systematically collect or analyse data on missing incidents or related harm outcomes. This lack of regular review creates a significant gap in understanding the scope and nature of missing incidents involving persons living with dementia. To address this gap, there is a pressing need for a comprehensive overview of these cases from a Swedish perspective, in order to identify the factors that increase the risk of harm [33]. Hence, the aim of this study was to describe missing incidents and explore factors associated with harm in persons living with dementia reported to the Swedish Police.

## Method

### Design

This nationwide retrospective registry study used police data from the Swedish STORM registry collected between 1 October 2021 and 30 September 2022.

### Setting and STORM registry

Sweden’s national police organization is divided into seven police regions, each with its own command centre. When the public requires urgent police assistance and calls the emergency number 112, these calls are directed to one of these command centers, for example, calls concerning missing persons. All calls are documented by a police operator as case reports in the record management system, STORM (System for Tasking and Operational Resource Management). Each case report is categorized into one of a wide range of categories, one of which is “missing person”. The case report also includes case specific information, urgency priority, time stamps, police patrol activities, harm outcome, place missing from, and background characteristics of the missing person such as sex and age. Most information is recorded as free text, including harm outcome or details regarding the presence of dementia, or by using a drop-down menu selected by the police operator, based on information provided by the caller or the police patrol. Information related to time stamps and police patrol activities is automatically logged into the system. In 2022, 1.1 million case reports were registered in the STORM registry (Swedish Police Authority, 2023). During the study period, a total of 27,286 missing person case reports were registered.

### Sample

**Fig. 1:**
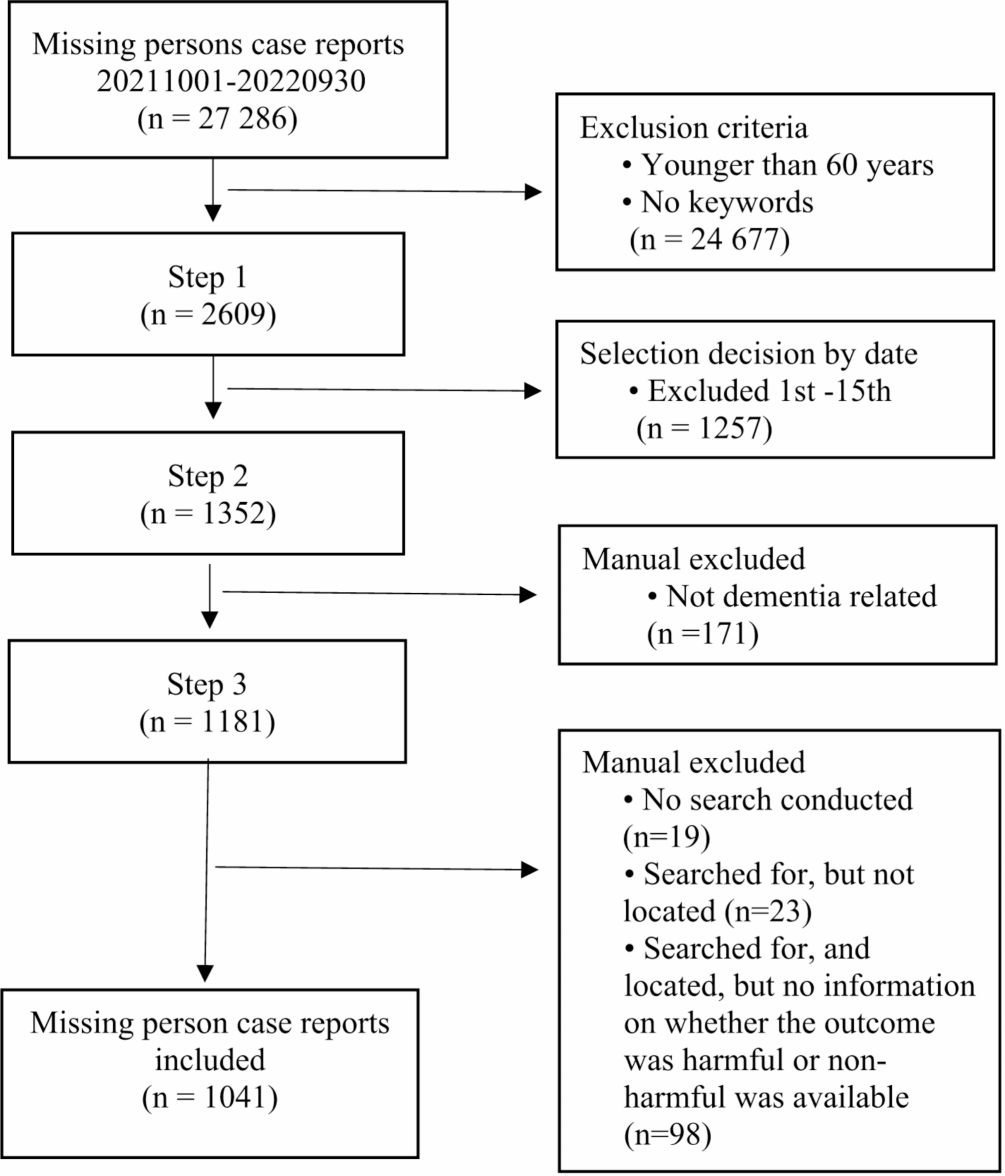
Sample selection process for missing person case reports from the STORM registry

The study sample was drawn from the STORM registry; one of the researchers (ML) was granted access to the registry and its content. However, due to technical constraints, access was limited to data less than 13 months old. The sampling process is detailed in Fig. 1. To be included, persons had to be 60 years or older, have a documented diagnosis of dementia in the STORM registry, and include clear information in the free text about whether the outcome was harmful or non-harmful. Although specifying the type of harm was not required, it was documented when provided. Harm was defined as requiring a visit to a healthcare facility for care or evaluation, or as being found deceased. In this study, a person living with dementia was defined as someone perceived as having dementia based on an overall assessment by the police. This did not necessarily require a clinical diagnosis but could be inferred from statements indicating that the individual was living with dementia, that dementia was suspected, or that a dementia assessment was pending.

To identify missing person case reports that fulfilled the inclusion criteria, persons aged 60 years or older who were reported missing between October 2021 and September 2022 were first identified. The reports were then further filtered to include only those that also mentioned the keywords “dementia” or “Alzheimer’s” (in Swedish), ensuring that all missing person case reports matched the age, time period, and cognitive condition criteria. Due to access being restricted to a single researcher and the technical limitations outlined, the initial sample size proved too extensive for a single researcher to manage. To ensure representation across all months of the year, the sample was limited to reports filed from the 16th to the last day of each month. After this, the remaining missing person case reports were manually screened for the inclusion criteria. Reports missing data on harmful or non-harmful outcomes, typically due to documentation omissions as well as reports indicating the absence of a search effort or failure to locate the missing person were excluded. The final selection included 1,041 missing person case reports.

### Study variables

For the present study, data on background characteristics, missing incident characteristics, and police response were taken from the STORM registry. Background characteristics included sex, age, and prior missing incidents. Data on the missing incident collected included place from which the person went missing, who reported the missing incident to the police, time of day for the incident, time of the year, time since last contact, mode of travel, who recovered the missing person, place of recovery, and if the person was harmed or not. Collected police response data included police urgency priority, if a police patrol was dispatched, and duration of the police search effort.

### Data analysis

Descriptive statistics were used to describe sample characteristics and study variables. Categorical data was presented using frequencies, continuous normally distributed data with means and standard deviations (SD), and continuously skewed distributed data with medians and percentiles (P_25_ and P_75_).

To explore factors associated with harm, a series of binary logistic regression analyses were conducted in a two-step approach. In the first step, simple binary logistic regression analyses were performed to explore the unadjusted association between each of the explanatory variables and harm (0 no harm; 1 harm). Thus, the simple binary logistic model served as a baseline model, which can facilitate the understanding of the association between each explanatory variable and the outcome variable. The explanatory variables covered three types of variables: background characteristics, missing incident characteristics, and police response. Background characteristics included sex (0 female; 1 male), age (years), and prior missing incidents (0 no; 1 yes; 2 unknown). Missing incident characteristics included who reported the incident (0 family member or friend; 1 care worker/healthcare professionals; 2 other), place missing from (0 ordinary living; 1 special housing for older people; 2 healthcare facility; 3 public place; 4 other), time of day (0 daytime; 1 afternoon/evening; 2 nighttime; 3 unknown), time since last contact (0 0–1:59 h; 1 2–3:59 h; 2 4–7:59 h; 3 ≥ 8 h; 4 unknown), time of the year (0 warm season; 1 cold season), mode of travel (0 on foot; 1 public transport; 2 by car; 3 unknown), Police response included police urgency priority (0 immediate; 1 urgent; 2 non-urgent), whether police patrol was dispatched (0 no; 1 yes), and duration of the police search effort (hours).

In the second step, a multiple binary logistic regression analysis was conducted, including all explanatory variables. The results from the binary logistic regression models are presented as odds ratios (OR) with corresponding 95% confidence intervals. The goodness of fit of the multiple regressions model was examined by the Hosmer and Lemeshow test (χ² [8] = 5.82, *p* = 0.670) and Nagelkerke R Square (0.31), both indicating acceptable model fit. In addition, no problems with multicollinearity were identified according to the variance inflation factor (VIF < 2).

Statistical significance was set at *p* < 0.05 and data were analysed using IBM SPSS statistics 28 (IBM Corp., Armonk, New York, United States).

## Result

### Background characteristics

Of the 1,041 missing person case reports analysed in this study, 602 (58%) involved males, yet this group represented less than 48% of the population aged 60 and above in Sweden [35]. The mean age was 79.3 (SD = 7.5; range = 60–101) years. A majority had previously gone missing, 84% vs. 16% (Table 1).

**Table 1:** Sample characteristics and study variables

	Total (*n* = 1041) ^a^	No harm (*n* = 980) ^b^	Harm (*n* = 61) ^b^
*Background characteristics*			
Age, mean (SD), [min– max]	79.3 (7.5) [60–101]	79.3 (7.5) [60–101]	79.2 (6.3) [61–90]
Sex, n (%)			
Female	439 (42.2)	422 (96.1)	17 (3.9)
Male	602 (57.8)	558 (92.7)	44 (7.3)
Previous missing incident, n (%)			
Yes	438 (84.0)	419 (95.7)	19 (4.3)
No	84 (16.0)	70 (83.3)	14 (16.7)
Unknown	519	491	28
*Missing incident characteristics*			
Reported missing by, n (%)			
Family member or friend	416 (40.0)	374 (89.9)	42 (10.1)
Care worker/healthcare professional	603 (57.9)	586 (97.2)	17 (2.8)
Other	22 (2.1)	20 (90.9)	2 (9.1)
Place missing from, n (%)			
Ordinary living	442 (42.5)	402 (91.0)	40 (9.0)
Special housing for older adults	410 (39.4)	399 (97.3)	11 (2.7)
Healthcare facility	60 (5.8)	59 (98.3)	1 (1.7)
Public place	58 (5.6)	54 (93.1)	4 (6.9)
Other	71 (6.8)	66 (93.0)	5 (7.0)
Time of the day, n (%)			
Morning (05:01–13:00)	248 (23.8)	238 (96.0)	10 (4.0)
Afternoon (13:01–21:00)	543 (52.2)	512 (94.3)	31 (5.7)
Nighttime (21:01–05:00)	148 (14.2)	137 (92.6)	11 (7.4)
Unknown	102 (9.8)	93 (91.2)	9 (8.8)
Hours since last contact, n (%)			
00:00–01:59 h	588 (56.5)	568 (96.6)	20 (3.4)
02:00–3:59 h	159 (15.3)	149 (93.7)	10 (6.3)
04:00–7:59 h	107 (10.3)	92 (86.0)	15 (14.0)
≥ 8 h	65 (6.2)	55 (84.6)	10 (15.4)
Unknown	122 (11.7)	116 (95.1)	6 (4.9)
Time of the year, n (%)			
Warm season (April-September)	610 (58.6)	584 (95.7)	26 (4.3)
Cold season (October-March)	431 (41.4)	396 (91.9)	35 (8.1)
Mode of travel, n (%)			
On foot	685 (65.8)	646 (94.3)	39 (5.7)
Public transport	29 (2.8)	28 (96.6)	1 (3.4)
Car (driver)	22 (2.1)	21 (95.5)	1 (4.5)
Unknown	305 (29.3)	285 (93.4)	20 (6.6)
Recovered by, n (%)			
Police	337 (32.4)	307 (91.1)	30 (8.9)
Care worker/healthcare professional	192 (18.4)	190 (99.0)	2 (1.0)
Family member or friend	201 (19.3)	196 (97.5)	5 (2.5)
Search and rescue organization or military	3 (0.3)	1 (33.3)	2 (66.7)
Other	308 (29.6)	286 (92.9)	22 (7.1)
Place of recovery, n (%)			
Own residence	52 (5.0)	52 (100)	0 (0)
Surrounding area	50 (4.8)	49 (98.0)	1 (2.0)
Public place outside	441 (42.4)	407 (92.3)	34 (7.7)
Public place inside	45 (4.3)	44 (97.8)	1 (2.2)
Returned on their own	151 (14.5)	151 (100)	0 (0.0)
Hospital	54 (5.2)	37 (68.5)	17 (31.5)
Previous residence	57 (5.5)	57 (100)	0 (0)
Hotspot	10 (1.0)	10 (100)	0 (0)
Other	181 (17.4)	173 (95.6)	8 (4.4)
*Police response*			
Police urgency priority, n (%)			
Non-urgent response	370 (35.5)	351 (94.9)	19 (5.1)
Immediate response	63 (6.1)	59 (93.7)	4 (6.3)
Urgent response	608 (58.4)	570 (93.8)	38 (6.3)
Police dispatched, n (%)			
No	338 (32.5)	323 (95.6)	15 (4.4)
Yes	703 (67.5)	657 (93.5)	46 (6.5)
Duration of police search effort (h), Mdn (P_25–_ P_75_) [Min–Max]	1.36 (0.74–2.56) [0.06–137.72]	1.31 (0.73–2.47) [0.06–19.54]	2.17 (1.10–5.88) [0.27–139.72]

^a^ Categorical data is presented as column percentage

^b^ Categorical data is presented as row percentage

### Missing incident characteristics

A description of the missing incidents is presented in Table 1. Most of the missing incidents (*n* = 603, 58%) were reported to the police by care workers or healthcare professionals. Persons living with dementia most frequently went missing from an ordinary living environment (*n* = 442, 43%) and most incidents occurred in the afternoon (*n* = 543, 52%) where the person living with dementia went missing on foot (*n* = 685, 66%) and were reported to the police within two hours (*n* = 588, 57%). Persons living with dementia were most frequently found in a public place outside (*n* = 441, 42%), typically walking in the streets or a walkway. One fifth (*n* = 203, 20%) returned home on their own or were found in their residence. Missing persons living with dementia were rarely located at a hotspot (*n* = 10, 1%). Hotspots are defined as locations where a missing person is likely to be found, based on an analysis of their personal history, routines, and significant places. Almost one third (*n* = 337, 32%) of the missing persons were located by the police, not only through the dispatching of patrols but also through information outreach efforts, such as when a police operator contacts the local hospital and discovers that the missing person had been admitted to a ward or was in the emergency department (Table 1).

### Police response

The police responded to the missing incidents by dispatching police patrol in 703 (68%) of all cases. Most cases (*n* = 608, 58%) required an urgent police response, defined as a response within 20 min according to police urgency protocol. The median duration of police search effort from the initial call to the police until the missing person case report was closed was 1.36 h with an interquartile range between 0.74 and 2.56 h (Table 1), the longest missing incident lasted 137 h. Most missing persons living with dementia (*n* = 980, 94%) were found unharmed while 61 (6%) were harmed (Table 1). Among those with a harmful outcome, four were found deceased. The most common cause of harm were injuries related to falls (*n* = 14), hypothermia (*n* = 10), lacerations (*n* = 8), or a poor general condition (*n* = 6), or a combination thereof. In 18 cases (30%), the type of harm could not be determined from the missing person case report.

### Factors associated with harm

The simple logistic regression showed that sex, prior missing incidents, who the person was reported missing by, place missing from, time since last contact, time of the year, and duration of police search effort were significantly associated with harm (Table 2). Being male (OR = 1.957, *p* = 0.022), not having previous missing incidents (OR = 4.411, *p* < 0.001), going missing during the cold season (OR = 1.985, *p* = 0.010) and prolonged duration of police search effort (OR = 1.220, *p* < 0.001) were associated with a higher probability of harm. In addition, a longer duration between last contact with the missing person and the call to the police, both 4–7:59 h (OR = 4.63, *p* < 0.001) and more than 8 h (OR = 5.164, *p* < 0.001), significantly increased the probability of harm compared to calls within two hours of going missing. Being reported as missing by care workers or healthcare professionals was associated with a significant lower probability of harm (OR = 0.258, *p* < 0.001) compared to being reported as missing by family or friends. This was also the case when persons living with dementia were reported as missing from a special housing for older people (OR = 0.277, *p* < 0.001), compared to ordinary living (Table 2). The duration of the police search effort had an exponential impact, with the probability of a harm outcome increasing by the hour (OR = 1220, *p* < 0.001).

**Table 2:** Factors associated with harm in missing persons living with dementia

	Simple binary logistic regression			Multiple binary logistic regression ^a^		
Explanatory variables	OR	95% CI	p-value	OR	95% CI	p-value
*Background characteristics*						
Sex, male	1.957	1.103–3.474	0.022	2.138	1.092–4.184	0.027
Age	0.998	0.964–1.034	0.928	1.006	0.963–1.051	0.787
Previous missing incident						
Yes	Ref			Ref		
No	4.411	2.114–9.201	< 0.001	4.949	2.001–12.241	< 0.001
Unknown	1.258	0.692–2.285	0.452	1.504	0.750–3.015	0.250
*Missing incident characteristics*						
Reported missing by						
Family member or friend	Ref			Ref		
Care worker/healthcare professional	0.258	0.145–0.461	< 0.001	0.449	0.193–1.044	0.063
Other	0.890	0.201–3.944	0.879	0.920	0.169–4.996	0.923
Place missing from						
Ordinary living	Ref			Ref		
Special housing for older adults	0.277	0.140–0.548	< 0.001	0.682	0.253–1.839	0.450
Healthcare facility	0.170	0.023–1.262	0.083	0.516	0.061–4.343	0.543
Public place	0.744	0.256–2.162	0.588	1.048	0.269–4.080	0.946
Other	0.761	0.290–1.999	0.580	0.981	0.323–2.976	0.973
Part of day						
Morning (05:01–13:00)	Ref			Ref		
Afternoon (13:01–21:00)	1.441	0.695–2.988	0.326	2.156	0.905–5.137	0.083
Nighttime (21:01–05:00)	1.911	0.791–4.615	0.150	2.165	0.767–6.111	0.145
Unknown	2.303	0.907–5.849	0.079	1.874	0.509–6.903	0.345
Hours since last contact						
0:00–1:59	Ref			Ref		
2:00–3:59	1.906	0.874–4.159	0.105	1.454	0.589–3.588	0.417
4:00–7:59	4.630	2.288–9.369	< 0.001	4.941	2.053–11.891	< 0.001
≥ 8	5.164	2.302–11.584	< 0.001	3.247	0.971–10.865	0.056
Unknown	1.469	0.577–3.738	0.420	1.592	0.497–5.093	0.434
Part of the year						
Warm season (April-September)	Ref			Ref		
Cold season (October-March)	1.985	1.176–3.350	0.010	3.032	1.570–5.858	< 0.001
Mode of transport						
On foot	Ref			Ref		
Public transport	0.592	0.078–4.462	0.611	0.403	0.042–3.861	0.431
Car (driver)	0.789	0.103–6.017	0.819	0.401	0.048–3.370	0.400
Unknown	1.162	0.666–2.028	0.596	0.566	0.277–1.158	0.119
*Police response*						
Police urgency priority						
Non-urgent response	Ref			Ref		
Immediate response	1.252	0.412–3.812	0.692	1.111	0.274–4.510	0.883
Urgent response	1.232	0.699–2.170	0.471	1.696	0.767–3.750	0.192
Police dispatched						
No	Ref			Ref		
Yes	1.508	0.829–2.741	0.178	0.646	0.276–1.510	0.313
Duration of police search effort (h)	1.220	1.143–1.307	< 0.001	1.260	1.150–1.380	< 0.001

aThe multiple logistic regression model was controlled for police region but are not included in the table

In the multiple binary logistic regression analysis, most of the significant associations from the simple logistic regression remained. However, the reporting person, previously identified as care workers or healthcare professionals, and the place missing from, which had been special housing for older people, were no longer significant. Time since last contact was still significantly associated with harm, but only regarding those with a time of four to eight hours compared to less than two hours since last contact (OR = 4.94, *p* < 0.001) (Table 2).

## Discussion

This study describes missing incidents reported to the Swedish Police through missing person case reports and explores factors associated with harm in persons living with dementia. As far as we know, this is the first study of its kind in a Swedish or Nordic context. The three main findings can be summarized as being the high number of missing person case reports involving persons living with dementia, the increased risk of harm within this group, and the importance of a swift police search effort.

Persons living with dementia represent a substantial portion of missing person case reports filed with the Swedish Police. Previous research supports that persons living with dementia are at an increased risk of going missing [30, 36–39]. In this study, 1,181 missing person case reports involving this group were identified during step 1–3 in the sampling process. Based on these findings, it is estimated that approximately 2,400 persons living with dementia were reported missing over the entire study period. In contrast, roughly 17,000 within this group are reported missing to the Japanese police each year [24], in a country with an estimated 5 million persons living with dementia [40]. Japan, unlike many other countries, maintains national statistics on missing persons, making it a valuable point of comparison. Given that there are an estimated 150,000 persons living with dementia in Sweden [11], this group is approximately five times more likely to be reported missing to the police compared to their counterparts in Japan. The reasons for this may be numerous and fall outside the scope of this study. Previous estimates from the Swedish Police indicated that persons living with dementia comprised approximately 20% of all missing person case reports [33]. The current study, however, revises this estimate downward to 9%. This discrepancy may arise from the Swedish Police Authority’s tendency to generalize findings from search and rescue operations to encompass all police search efforts, potentially leading to an overgeneralization of the data.

The current study found that nearly one-fifth of persons living with dementia reported missing returned independently or were located at home, suggesting they may have been momentarily elsewhere or out of sight rather than truly lost. This aligns with findings from studies in the UK and South Korea [30, 41]. Like previous research, this study also found that repeat missing incidents were common, with a prior incident increasing the risk of future occurrences [30, 38, 41, 42]. Additionally, a greater proportion of missing incidents involved persons living with dementia in specialized housing for older people compared to findings from other studies [19, 30, 43]. However, due to the selection process used, this requires further investigation for a proper comparison. Also, care arrangements for persons living with dementia vary between countries; the majority of persons living with dementia reside in low- and middle-income countries, where care is primarily family-based due to the prohibitive cost of public or private care options [44]. This makes direct comparisons with Sweden difficult. In Sweden, while family are also primary caregivers, the welfare system ensures access to professional care for all, regardless of financial circumstances [45]. Municipal care in Sweden is considered high quality and is widely accepted when needed.

Most of the missing incidents in this study were reported by care workers or healthcare professionals, highlighting the importance of improving collaboration among professional stakeholders to enhance safety and reduce the number of missing incidents. Strengthening information-sharing strategies in the care of persons living with dementia could reduce miscommunication and misunderstanding, thereby lowering the number of missing persons reports to the police. Furthermore, improved information-sharing between professional caregivers and the police, through access to critical information, can enhance the efficiency of police search efforts and expedite the recovery process.

Persons living with dementia face a significant risk of harm when missing. This study found that more than one in twenty reported missing were harmed. Increased risk of harm has been observed in previous studies as well, though the proportion varies depending on the sampling and the definition of harm used [19, 24, 26, 30, 41]. In this study, harmful outcomes were associated with the need for care or evaluation at a healthcare facility, posing a significant risk that other forms of harm, such as psychological or emotional harm, may have been overlooked [46, 47]. These harmful outcomes can manifest as confusion, stress, fear, or anxiety for a person living with dementia and can lead to increased monitoring, reduced autonomy, and decreased well-being. Most of these harmful outcomes can be experienced by the caregiver as well, along with heightened strain and a feeling of guilt [6, 22, 48]. Although most injuries in this study were minor, the range in severity was wide, with four persons living with dementia found deceased. Hospitalization due to falls in this group is associated with more adverse outcomes, longer hospital stays, and higher mortality rates compared to those living without dementia [49]. Minor injuries in this group, such as lacerations or wounds, can be challenging to treat successfully, increasing the risk of infection, pain, and mobility issues, which may result in more serious complications [50].

Several factors were significantly associated with an increased probability of harm, which included male sex, first time missing incident, cold season, delayed reporting, and prolonged police search effort. The current study showed that the duration of the police search effort emerged as the strongest predictor of harm outcome. The search effort is the only factor that can be affected by the police directly. The importance of time has been demonstrated in numerous previous studies [5, 19, 20, 28, 29, 51]. However, this study used hours as the time variable rather than the more common measure of days. Since persons living with dementia tend to behave unpredictably when disoriented, Rowe, Feinglass and Wiss [52] argue, based on their case study and review of research, that a search effort should be conducted systematically using general search guidelines rather than relying on specific tips or leads, as these have often proven to be inaccurate. The search should start as quickly as possible, with resources and actions gradually increasing. Most missing persons living with dementia do not move far from where they were last seen. Therefore, if the missing person is not found within a reasonable timeframe, the search effort should still focus on the area within one or a few kilometers, with particular attention to less accessible locations such as wooded areas, bushes, ditches, and natural water sources as persons living with dementia may seclude themself rather than seek help.

It’s being the first time a person living with dementia went missing was associated with increased probability of harm. This is consistent with the findings by Doyle and Barnes [26] in their study from England, who also observed an increased probability of harm when older adults went missing for the first time. Perhaps the risk of harm is more difficult to assess the first time a person living with dementia goes missing and there may be greater hesitation to contact the police during the initial incident, potentially delaying the response. Additionally, it could have been more difficult for the police to obtain the crucial information needed for an effective response, further complicating the situation. The cause of the increased probability of harm in such cases warrants further investigation to better understand these dynamics. To further enhance safety and reduce the consequences of persons living with dementia going missing, a multi-professional and multi-stakeholder approach is necessary [3]. Professional caregivers often lack the necessary information requested or take actions in an incorrect order, according to the police. Conversely, the police may lack adequate knowledge about dementia, including its signs and symptoms. A closer collaboration and exchange of knowledge and experience could benefit all parties, especially persons living with dementia [3, 39].

### Strengths and limitations

This study has both strengths and limitations. A key strength is the large sample that covers all police regions in Sweden. This extensive coverage enhances the robustness of the findings and reduces the risk of selection bias, providing a more comprehensive understanding of missing incidents in persons living with dementia across Sweden and factors associated with harm. However, this study also has some limitations. First, this study evaluated the presence of dementia in the missing person based on an overall assessment made by the police, rather than through medical evaluations or patient records. Although the information primarily came from family members and professional caregivers, there is a possibility that persons who were mistakenly identified as living with dementia may have been included in the study. A second limitation was the reliance on keywords as a selection variable, which may have resulted in the unintentional exclusion of missing persons case report from the study due to misspellings or variations in terminology. A third limitation was the exclusion of missing person case reports where the person living with dementia was either not searched for or searched for but not located. This may have excluded missing incidents that resulted in harmful outcomes, potentially giving a misleading picture of the actual proportion of persons living with dementia who came to harm when missing.

## Conclusion

This study demonstrates that the risk of harm is significant among missing persons living with dementia. Given that these persons are particularly vulnerable, there is a risk that even minor injuries can have substantial consequences. This study identified several factors associated with an increased probability of harm that are crucial to consider in risk assessment, urgency prioritization, and the allocation of search resources. One factor the police can influence is the duration of the search effort, and there are considerable benefits for both the persons living with dementia and society if this duration can be reduced. This study underscores the need for policy measures that promote shared responsibility among multiple actors, including the police. It also calls for the development of collaborative routines and action plans between the police and professional caregivers caring for persons living with dementia to be implemented before any potential missing incidents in order to facilitate an effective police search effort.

## Data Availability

Data are available from the corresponding author on reasonable request.
